# Fracture of Upper Molar by a Fall

**Published:** 1883-01

**Authors:** C. Vincent Cotterell

**Affiliations:** Dental Surgeon to St. Bartholomew's Hospital, Rochester.


### ARTICLE VIII.

### *Fracture of an Upper Molar Caused by a Fall.*

---

BY C. VINCENT COTTERELL., L. D. S., DENTAL SURGEON TO ST. BARTHOLOMEW'S HOSPITAL, ROCHESTER.

---

Writes Colonel H. W,—of the Royal Engineers, whilst stationed at Chatham in 1852, thirty years ago, was attacked with acute periostitis in the upper wisdom tooth of the left side through cold, and attributing the pain to caries, consulted his friend, a medical man, who immediately extracted the tooth. As there was a total absence of caries, the inflammation was assigned to the rheumatic diathesis.

Ten years afterwards in attempting to mount a restive horse he vaulted over its back and fell heavily on the grass, striking his right shoulder and cheek. On regaining his feet he imagined he had dislocated his shoulder and broken his jaw, which happily the army surgeon assured him was not the case. Great difficulty was experienced for many days in opening the mouth owing to stiffness of the muscles, but beyond that no dental disturbance took place. About eight years elapsed, when a piece of bone worked out of the place where the wisdom tooth was extracted, which the army surgeon said was a piece of alveolus.

In 1881 he consulted me for advice respecting an offensive taste proceeding from the region of the second upper molar of the left side, and also produced the so-called piece of alveolus which I found was the root of a tooth. After a careful examination with my probe I passed it into a cavity, situated on the distal surface and about the eighth of an inch above the neck of the tooth. As the tooth was loose, and the discharge caused the patient considerable annoyance, I extracted it, when I found the posterior buccal fang was missing, and when the root, which had worked out eight years previously, was put to the broken surface it articulated most perfectly, showing clearly that it belonged to the tooth just extracted. The discharge immediately ceased, which has led me to the conclusion that the tooth was broken by the fall, and the pulp had retained its vitality for some years, its decomposition taking place just prior to the appearance of the discharge—*British Journal of Dental Science.*

